# Brain-dead and coma patients exhibit different serum metabolic profiles: preliminary investigation of a novel diagnostic approach in neurocritical care

**DOI:** 10.1038/s41598-021-94625-3

**Published:** 2021-07-30

**Authors:** Tomasz Dawiskiba, Wojciech Wojtowicz, Badr Qasem, Marceli Łukaszewski, Karolina Anna Mielko, Agnieszka Dawiskiba, Mirosław Banasik, Jan Paweł Skóra, Dariusz Janczak, Piotr Młynarz

**Affiliations:** 1 Department of Vascular, General and Transplantation Surgery, Wroclaw Medical University, Ul. Borowska 213, 50-556 Wroclaw, Poland; 2 Department of Biochemistry, Molecular Biology and Biotechnology, Faculty of Chemistry, Wroclaw University of Science and Technology, Norwida 4/6, 50-373 Wroclaw, Poland; 3 Department of Anesthesiology and Intensive Therapy, Wroclaw Medical University, Ul. Borowska 213, 50-556 Wroclaw, Poland; 4 Department of Nephrology and Transplantation Medicine, Wroclaw Medical University, Ul. Borowska 213, 50-556 Wroclaw, Poland

**Keywords:** Biomarkers, Medical research, Neurology

## Abstract

There is a clear difference between severe brain damage and brain death. However, in clinical practice, the differentiation of these states can be challenging. Currently, there are no laboratory tools that facilitate brain death diagnosis. The aim of our study was to evaluate the utility of serum metabolomic analysis in differentiating coma patients (CP) from individuals with brain death (BD). Serum samples were collected from 23 adult individuals with established diagnosis of brain death and 24 patients in coma with Glasgow Coma Scale 3 or 4, with no other clinical symptoms of brain death for at least 7 days after sample collection. Serum metabolomic profiles were investigated using proton nuclear magnetic resonance (NMR) spectroscopy. The results obtained were examined by univariate and multivariate data analysis (PCA, PLS-DA, and OPLS-DA). Metabolic profiling allowed us to quantify 43 resonance signals, of which 34 were identified. Multivariate statistical modeling revealed a highly significant separation between coma patients and brain-dead individuals, as well as strong predictive potential. The findings not only highlight the potential of the metabolomic approach for distinguishing patients in coma from those in the state of brain death but also may provide an understanding of the pathogenic mechanisms underlying these conditions.

## Introduction

In classical terms, human death as a whole has been identified by the irreversible cessation of breathing and blood circulation^1,2^. Shifting the boundary of death and extending it to patients with critical brain damage was revolutionary and changed the paradigms of modern medicine. It is assumed that it is impossible to determine either the beginning or the end of death, but medical criteria that most certainly confirm the irreversibility of this phenomenon should be provided at any time in its duration. The diagnosis of brain death in most cases can be based only on clinical examination with a detailed analysis of the causes, mechanisms and effects of brain damage^3–6^. Situations in which clinical tests allowing the diagnosis of brain death cannot be executed or clearly interpreted include cases of subtentorial brain damage, cases of extensive facial injuries, situations with the presence of abnormal neurological reflexes, and cases of being under the influence of certain contaminants or specific pharmacological agents^2,7^. The diagnostic process must then be complemented by additional tests such as cerebral arteriography, which are not always easily accessible. It should be noted that laboratory diagnostics still have not proven to be a useful tool in such situations. Recognition of brain death, or at least suspicion of brain death, could resemble in such cases the diagnosis of acute myocardial infarction with the fundamental role of serum troponin determination. So far, the presumed potential of only two proteins has been initially disclosed S100B protein^8–10^ and neuron-specific enolase NSE^9,11^, but these are single reports only, and no comprehensive analysis applying modern proteomic methods has yet been performed. It can therefore be concluded that, despite the seriousness of the problem, this theme is a kind of terra incognita in medicine, scientific reports dedicated to this subject are practically nonexistent, and the search for new methods of brain death diagnosis seems to be an absolute necessity.

The concept of metabolomic studies is based on the observation that with the development of pathological processes, both local and systemic, the first symptoms appearing at the cellular level are directly reflected in the chemical composition of tissues subject to these processes and also in body fluids. These processes may be minor changes involving the disruption of the quantitative ratio between different chemical compounds as well as changes that are easier to detect, such as the appearance of entirely new chemical compounds (biomarkers of disease) or the disappearance of specific molecules present in homeostasis. The analysis of the abovementioned issue allows for the creation of predictive and discriminatory models by which the detection of even subtle changes in the concentrations of metabolites constitute the differentiating factor^12–14^. Metabolomics research is primarily based on gas or liquid chromatography–mass spectrometry (GC–MS/LC–MS) and nuclear magnetic resonance (NMR) platforms^12–15^.

The aim of our research was to answer the question of whether there are changes in the profile of low molecular weight compounds present in blood serum in the process of brain death and to identify the metabolic biomarkers of this condition. This is the first such report in the literature.

## Materials and methods

### Serum sample collection

Serum samples of brain-dead (BD) individuals and coma patients (CP) were collected mainly at the Department of Anesthesiology and Intensive Therapy of Wroclaw Medical University. Additionally, biological material from brain-dead cadavers was collected in other hospitals in the region of Lover Silesia during organ procurements performed by surgeons of the Department of Vascular, General and Transplantation Surgery of Wroclaw Medical University (the same protocol). The study was approved by the Commission of Bioethics at Wrocław Medical University (Approval no. KB-25/2018), and written informed consent was obtained from legal representatives of all the patients before enrollment in the study. All these research were conducted according to The Code of Ethics of the World Medical Association (Declaration of Helsinki).

The samples were collected from March 2018 to April 2019 from adult individuals with established diagnosis of brain death (n = 23) and comatose patients (n = 24) (Table 1). The diagnosis of brain death was determined and certified by three independent specialists according to current Polish law^16,17^ and was not related to the research project. The control group was formed of patients in coma in Glasgow Coma Scale 3 or 4, with no other clinical symptoms of brain death for at least 7 days after collection of serum (preservation of brain stem reflexes and/or of respiratory drive). No analgosedation was applied in the enrolled patients. The primary causes of brain death and coma were hypoxic-ischemic brain injury, traumatic brain injury and nontraumatic intracerebral hemorrhage (detailed information in Table 1). Patients with other causes of death or coma (e.g., brain tumors or meningitis) were excluded from the study, as well as those below 18 years, cases with evidence of malignancy or xenobiotic intoxication and individuals subjected to renal replacement therapy. No pregnant women were enrolled in the study. Signals related to medications (mannitol, furosemide, proton-pump inhibitors, antibiotics, noradrenaline, steroids, low molecular weight heparins and antipyretics) were eliminated from the statistical and chemometric evaluation.

**Table 1:** Demographic data and clinical profile of patients.

	Brain-dead individuals	Coma patients
Number of patients − overall	23	24
Hypoxic-ischemic brain injury	5	8
Traumatic brain injury	4	8
Nontraumatic intracerebral hemorrhage	14	8
GCS 3/GCS 4	23/none	13/11
7/30/90 days survival − number of patients	None	24/18/13
Sex (male/female)	17/6	14/10
Average age (mean/range)	49.3 (22–74)	56.7 (19–87)

Serum was sampled from the peripheral vein and collected using serum vacuum tubes (BD Vacutainer ref. 369032) that were then centrifuged at 1000 × rpm for 15 min at 4 °C. The samples were stored in Eppendorf-type tubes and kept at − 80 °C until analysis. Transport was accomplished with the use of liquid nitrogen storage dewars.

### Samples preparation and NMR measurements

The collected serum samples were prepared according to a well-established protocol^18,19^. The serum samples were thawed at room temperature and vortexed. Each serum sample (200 μL) was mixed with 400 μL of saline solution (0.9% NaCl, w/v) containing 20% D_2_O and centrifuged (10 min, 12 000 RPM, 4 °C). Supernatant (550 μL) from each sample was transferred into a 5-mm NMR tube (SP, 5 mm ARMAR Chemicals). The samples were kept at 4 °C before measurement.

The one-dimensional (1D) NMR spectra of serum samples were recorded at 298 K using an Avance II spectrometer (Bruker, GmBH, Germany) and *cpmg1dpr* pulse sequence with water presaturation (*Bruker notation*), which was operating at a proton frequency of 600.58 MHz. The serum sample spectra were collected as 128 following scans with spin-echo delay of 1000 μs, 80 loops, relaxation delay of 3.5 s, acquisition time of 2.73 s, size of FID (TD), 65,536 points, spectra width of 20.01 ppm, line-broadening factor (LB), 0.3 Hz and transmitter frequency offset (O1P), 4.722 ppm.

Two-dimensional (2D) NMR experiments were recorded and processed for selected samples. The performed experiments included ^1^H−^1^H correlation spectroscopy (COSY), total correlation spectroscopy (TOCSY), and ^1^H−^13^C heteronuclear single quantum correlation (HSQC).

### Processing of NMR spectra and resonance signal identification for data analysis

The collected 1D ^1^H NMR spectra were processed with LB of 0.3 Hz and manually phased and baseline corrected with MestReNova software (Mestrelab Research v 12.0.4). The spectral chemical shifts were referenced on the glucose anomeric carbon signal group δ = 5.225 ppm. Spectral processing was performed in the 0.500 ppm–10.000 ppm chemical shift range. Spectral sections from 4.400 to 5.000 ppm, corresponding to water resonance signal suppression, were removed from the data matrix. The alignment of resonance signals was carried out with the use of the correlation optimized warping algorithm (COW)^20^ and the *icoshift* algorithm implemented in MATLAB (v R2019a, Mathworks Inc.)^20^. All of the spectra were normalized by the PQN (probabilistic quotient normalization) method^21^. The calculation of the relative integral of NMR measured metabolites was obtained as a sum of data points of the nonoverlapping resonances or a cluster of partly overlapping resonances from the data matrix consisting of 46,842 data points for each spectrum in n dimensions. The third quartile values of the noise region (0.625 ppm) were subtracted from the calculated relative integrals to decrease the influence on the final values.

The ^1^H NMR resonance signals and corresponding chemical shifts were analyzed with statistical total correlation spectroscopy (STOSCY)^22^ and identified in accordance with assignments published in the literature. Chenomx software (v 8.4 Chenomx Inc. Edmonton, Alberta, Canada) and online databases: Biological Magnetic Resonance Data Bank^23^ (www.bmrb.wisc.edu) and Human Metabolome Data Base^24^ (www.hmdb.ca).

### Univariate and multivariate data analysis

All univariate data analyses were carried out with MATLAB software (v R2019a, Mathworks Inc.) on non-scaled data. Values below the limit of quantification were replaced with the third quartile value of the noise region (0.625 ppm) for a specific variable. Levene’s test was used to assess homogeneity of variation. Normality of distribution was verified with the Shapiro–Wilk test. Depending on the results of normality and variance tests, a parametric (equal/unequal variance Student’s t-test) or nonparametric (Mann–Whitney–Wilcoxon test) variant was calculated. Multigroup univariate analysis was performed with the Kruskal–Wallis test with Dunn-Sidak post hoc tests. The false discovery rate (FDR) based on the Benjamini–Hochberg procedure was applied for the tested variables. All univariate statistical tests were calculated at a significance level of α = 0.05. Dispersion of variables was represented by the coefficient of variation (CV) for normally distributed data or otherwise by the coefficient of quartile variation (QCV). Assessment of classifier performance was represented by ROC curve analysis using the *perfcurve* MATLAB (v R2019a, Mathworks Inc.) function.

The model calculations were performed using unit variance (UV) scaling for the relative integral values, while Pareto (Par) scaling was applied to a data matrix containing data from the entire spectrum. Both data sets were then used for calculations in the SIMCA 15.0.2.5959 program [Sartorius Stedim Data Analytics AB, 2018)]. Principal component analysis (PCA) was applied for data overview and for extreme outlier detection based on Hotelling's T2 range (99%). Discriminant analysis was performed by the partial least squares method (PLS-DA) for relative integral data (43 variables corresponding to the range of specific resonance signals) and orthogonal partial least squares (OPLS-DA) for whole spectra data (46,842 variables as matching data points for chemical shifts). OPLS-DA data visualizations are presented together with Hotelling's T2 range (95%) ellipse. The reliability of the PLS-DA and OPLS-DA models was assessed by analysis of variance of the corss-validated residuals (CV-ANOVA) at a significance level α = 0.05. Discrimination model score plots are also presented as cross-validated versions in the Supplementary Materials^25^. The prediction effectiveness of the PLS-DA model is presented by a receiver operating characteristic (ROC) curve with the use of the *perfcurve* function in MATLAB (v R2019a, Mathworks Inc.). For this purpose, the true negative rate and true positive rate were determined according to sample class assignation, applying the sevenfold cross-validated predicted values from the modeled observations using the YPredcv function for the PLS-DA model (implemented in SIMCA 15.0.2.5959 software, Sartorius Stedim Data Analytics AB, 2018).

## Results

**Figure 1:**
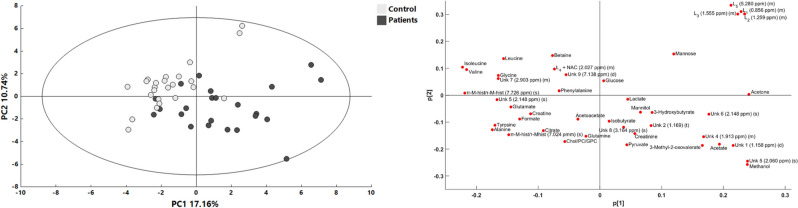
Principal component analysis (PCA) score plot (left) and corresponding loading plot (right) with all quantified ^1^H NMR variables for patients enrolled in the study. Light gray—coma patients (CP); gray—brain-dead individuals (BD).

**Figure 2:**
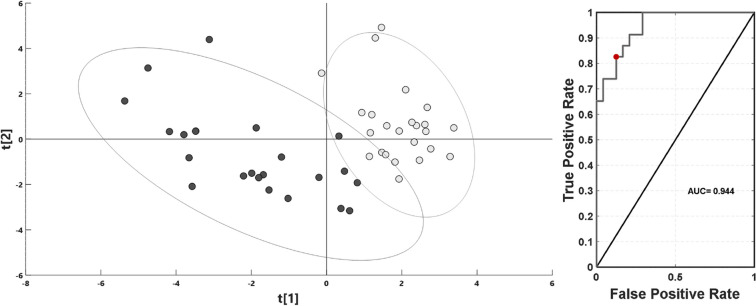
Partial least squares discriminant analysis (PLS-DA) score plot with two standard deviation range from mean for each group together with receiver operating characteristic curve (ROC) on first PLS-DA latent variable for sample classification. Light gray—coma patients (CP); gray—brain-dead individuals (BD); red circle on the ROC curve marks the optimal operating point.

**Figure 3:**
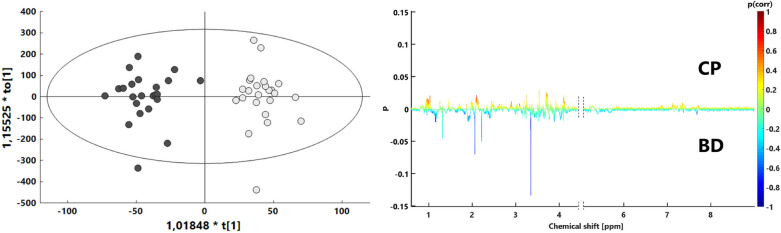
Orthogonal partial least squares discriminant analysis (OPLS-DA) score plot with corresponding s-line plot. Light gray—coma patients (CP); gray—brain-dead individuals (BD).

The ^1^H NMR spectra measurements allowed us to quantify 43 resonance signals: 34 identified and 9 unknown compounds. Two data types were used to assess the possible distinction between the studied groups: the first based on the abovementioned specifically selected quantified resonance signals (Figs. 1, 2) and the second with 46,842 data points (Fig. 3). Details of the chemical shifts and resonance signals taken for analysis are presented in the supplementary data (Table S1).

The data obtained were elaborated by univariate and multivariate analysis. The exploratory analysis that was accomplished with the use of a PCA model based on the relative integrals of resonance signals revealed spontaneous separation between the studied groups (CP vs BD), although its extent was not substantial. In addition, PCA based on the Hotelling T2 range with a significance level equal to 0.01 revealed no extreme outliers, enabling the use of all observations for discriminant analysis based on PLS and OPLS models. The variables that influenced the positions of observations in the PCA model are presented in Fig. 1.

Both calculated multivariate models demonstrated favorable separation between the CP and BD groups, attained high predictive potential and were strongly statistically relevant (Table 2). The PLS-DA model validation based on cross-validated analysis of variance (CV-ANOVA) revealed statistical significance with a p value = 1.56E−07, while the OPLS-DA model had a p value = 4.54E−05. The PLS-DA discriminant model was also assessed with the use of a receiver operating characteristic curve (ROC) and the area under the curve value (AUC). Its sensitivity and specificity were calculated from sample class prediction during the cross-validation procedure (YpredCV) in SIMCA v15.0.2.5959 software (Sartorius Stedim Data Analytics AB, 2018). The discriminant model obtained a high AUC value equal to 0.944. The graphical representations for PLS-DA and OPLS-DA comparisons between the CP and BD groups for the two latent variable models are shown in Figs. 2 and 3. To verify the influence of resonance signals that were not quantified, the OPLS-DA model based on whole-spectra analysis was calculated along with a graphical representation of the importance of the variables in the predictive component versus the chemical shift (Fig. 3, right). The graph is colored according to model loadings scaled as coefficients of correlation between the models and original data (Fig. 3, right).

**Table 2:** Main model parameters from PLS-DA for comparison between coma patients and brain-dead individuals.

Comparison	Model type	N	PC/LV	R2X (cum)	R2Y (cum)	Q2 (cum)	p value (CV-ANOVA)
CP vs BD (relative integral)	PCA	47	2	0.279	–	–	–
CP vs BD (relative integral)	PLS-DA	47	2	0.254	0.792	0.562	1.56E−07
CP vs BD (spectra)	OPLS-DA	47	1 + 4 + 0	0.671	0.914	0.613	4.54E−05

**Figure 4:**
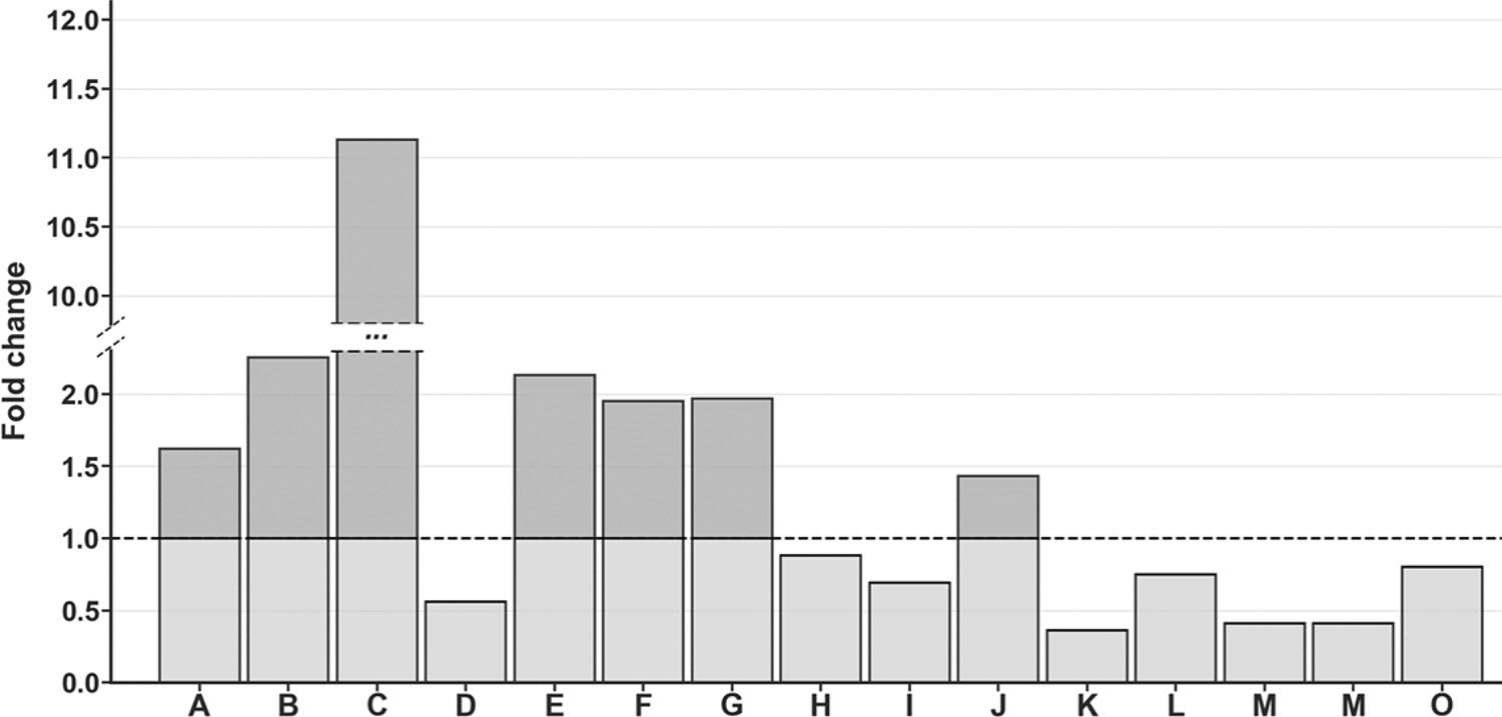
Relative integrals fold changes representation for brain-dead individuals vs coma patients among all statistically important metabolites in study. ^1^Student’s t-test, ^2^t-test for unequal variance, ^3^Mann-Whitney-Wilcoxon test. A^1^—Unk 1 (1.158 ppm) (d), B^2^—Unk 5 (2.060 ppm) (s), C^2^—methanol, D^2^—Unk 7 (2.903 ppm) (m), E^3^—acetone, F^3^—Unk 2 (1.169) (t), G^3^—acetate, H^3^—isoleucine, I^1^—betaine, J^2^—3-methyl-2-oxovalerate, K^3^—Unk 6 (2.148 ppm) (s), L^3^—methylhistidine (s), M^3^—glycine, N^3^—Unk 9 (7.138 ppm) (d), O^1^—valine.

**Figure 5:**
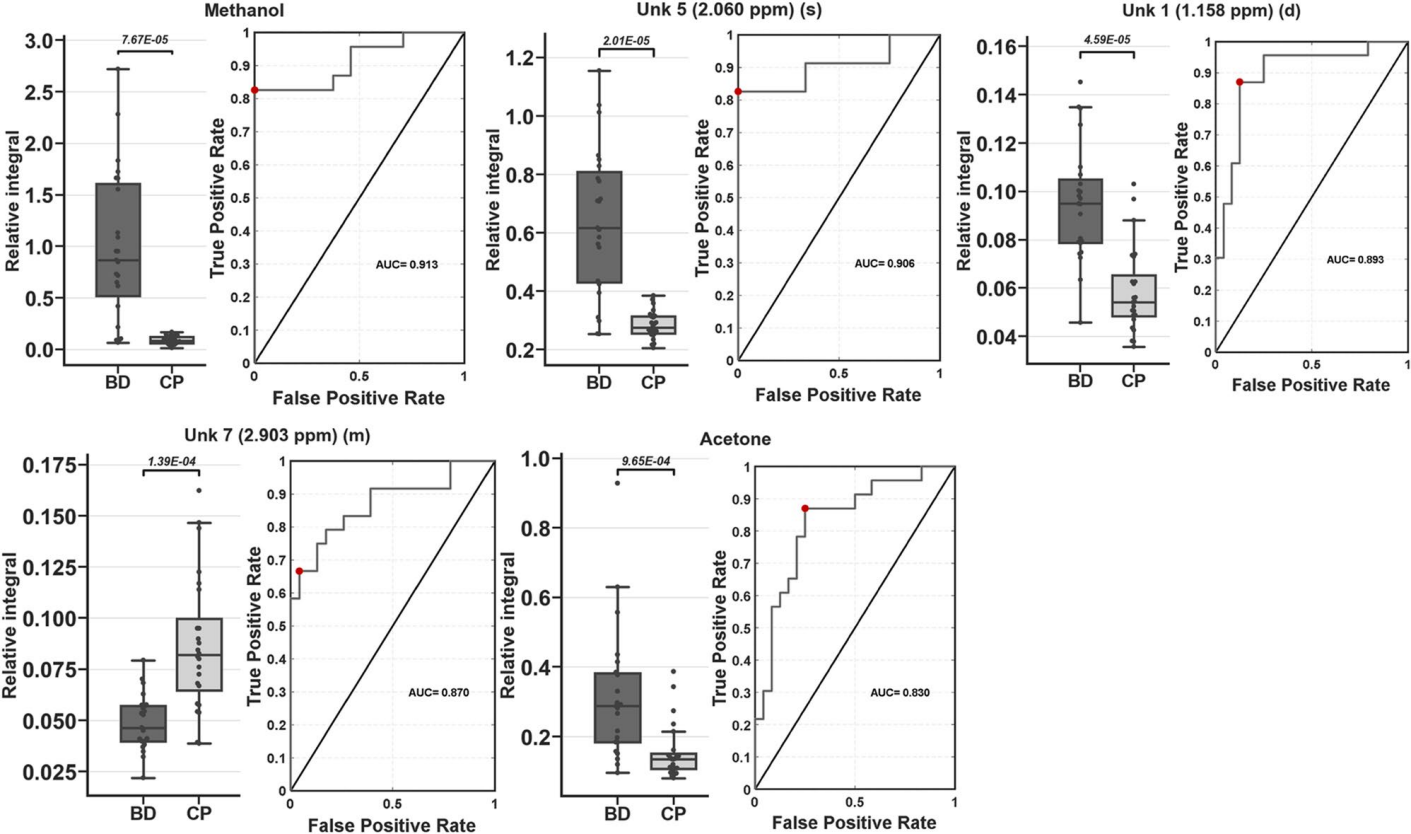
Boxplots and receiver operating characteristic curves (ROC) for statistically important metabolites with ROC area under curve (AUC) above 0.800 (AUC > 0.800). Boxplots are sorted by decreasing ROC AUC values. Statistical test used ^1^t-test for unequal variance, ^2^Student’s t-test or ^3^Mann–Whitney–Wilcoxon test: methanol^1^, Unk 5^1^ (2.060 ppm) (s), Unk 1^2^ (1.158 ppm) (d),—Unk 7^1^ (2.903 ppm) (m), acetone^3^. For boxplots: whiskers—1.5 × interquartile range (IQR); bar—average; box—range between first quartile (Q1) and third quartile (Q3). Red circle on ROC curve marking optimal operating point.

Verification of potentially important single variables as valuable classifiers between coma patients and brain-dead individuals proceeded with the use of univariate analysis. Statistically important metabolites are presented together with descriptive statistics and ROC curve (AUC) values (Table 3). Univariate analysis revealed significant differences in 15 resonance signals, of which 9 were identified according to chemical shift, STOCSY analysis and 2D NMR spectra, while 6 remained unidentified. For those statistically important metabolites, fold change values were calculated (Fig. 4), as well as their AUC values (Fig. 5). Seven metabolites were increased in the BD group (Unk 1, Unk 5, methanol, acetone, Unk 2, acetate, 3-methyl-2-oxovalerate), and 8 were decreased (Unk 7, isoleucine, betaine, Unk 6, methylhistidine, glycine, Unk 9 valine). The highest change was observed in the relative integral of the singlet resonance signal for methanol, with a fold change equal to 11.13. Of the abovementioned statistically important metabolites, 5 exhibited AUC values above 0.800 (Unk 1, Unk 5, methanol, Unk 7 and acetone), including 2 values even exceeding 0.900 (Unk 5 and methanol). The whole-spectra analysis by the OPLS-DA model confirmed the results of PLS-DA, indicating the two most important ^1^H NMR spectra resonance signals with p(corr) > 0.700 at 1.15 ppm and 3.34 ppm (corresponding to Unk 1 and methanol, respectively). The response permutation testing plots for the PLS-DA and OPLS-DA models are presented in the supplementary data (Fig. S1).

**Table 3:** Univariate analysis for quantified ^1^H NMR resonance signals compared between coma patients and brain-dead individuals.

Metabolite	Fold change (BD/CP)	CV or QCV (%) BD	CV or QCV (%) CP	FDR adjusted p-value	AUC
Unk 1 (1.158 ppm) (d)	1.62^(1)^	25.57^#^	30.51^#^	2.01E−05^a^	0.893
Unk 5 (2.060 ppm) (s)	2.25^(1)^	39.90^#^	16.79^#^	2.01E−05^b^	0.906
Methanol	11.13^(1)^	73.25^#^	44.00^#^	7.67E−05^b^	0.913
Unk 7 (2.903 ppm) (m)	0.56^(1)^	28.00^#^	38.03^#^	1.39E−04^b^	0.870
Acetone	2.13^(2)^	35.25*	18.05*	9.65E−04^c^	0.830
Unk 2 (1.169) (t)	1.95^(2)^	16.67*	23.75*	5.75E−03^c^	0.786
Acetate	1.97^(2)^	61.22*	39.90*	1.30E−02^c^	0.759
Isoleucine	0.88^(2)^	23.58*	18.36*	1.30E−02^c^	0.759
Betaine	0.69^(1)^	26.90^#^	43.51^#^	1.51E−02^b^	0.725
3-Methyl-2-oxovalerate	1.43^(1)^	37.20^#^	42.48^#^	1.51E−02^a^	0.754
Unk 6 (2.148 ppm) (s)	0.36^(2)^	44.73*	45.53*	1.51E−02^c^	0.745
Methylhistidine	0.75^(2)^	18.15*	12.29*	1.51E−02^c^	0.745
Glycine	0.41^(2)^	45.27*	47.42*	2.32E−02^c^	0.728
Unk 9 (7.138 ppm) (d)	0.41^(2)^	60.74*	52.23*	2.32E−02^c^	0.728
Valine	0.80^(1)^	24.78^#^	32.13^#^	3.50E−02^a^	0.745

The metabolite order is based on ascending adjusted *p* values. Calculations made on ^(1)^ mean or ^(2)^ median.

^#^CV (coefficient of variation).

*QCV (quantile coefficient of variation).

^a^t-test for equal variances.

^b^t-test for unequal variances.

^c^Mann–Whitney–Wilcoxon test.

## Discussion

Our ^1^H NMR-based metabolomic approach demonstrated serum metabolic differences between patients in coma and individuals with diagnosis of brain death. The results obtained showed favorable separation and model parameters in cross-validated PLS-DA and OPLS-DA models with high predictability in both relative integral and whole spectra data for the studied sample. The discrimination potential between groups was also confirmed for specific metabolites with high ROC AUCs. The findings not only establish the potential of metabolomics in neurocritical care but may also provide an understanding of the pathogenic mechanisms underlying brain death. This is the first scientific publication in the literature relating to metabolomic studies of brain death.

In recent years, a few research projects aimed at metabolomic assessment of clinical conditions associated with central nervous system pathologies have been conducted; the goal in most cases was to identify diagnostic biomarkers for stroke or traumatic brain injury. The use of multivariate statistical analysis has made it possible to demonstrate significant separation between patients with cerebral pathology and healthy individuals in most of the studies^15,26–37^. For example, in the case of stroke the group of potential biomarkers includes, among others: lactate^27,29,31,32^, pyruvate^27,29,32^, glycolate^27^, formate^27^, glutamine^27^, methanol^27^, acetate^29,32^, cysteine^26^, folic acid^26^, S-adenosyl homocysteine^26^, oxidized glutathione^26^, tyrosine^15,30,31^, tryptophan^31^, serine^30–32^, isoleucine^28,30^, valine^15,28,30,32^, glycine^32^, leucine^15,28^, betaine^30,32^, carnitine^15,30^ and ketone bodies (acetone, acetoacetate and β-hydroxybutyrate)^29^ as blood biomarkers; citrate^27^, hippurate^27^, and glycine^27^ as urine biomarkers; and finally acetic acid^38^, 3-hydroxyisovaleric acid^38^, 3-hydroxybutyric acid^38^, choline ^38^, glycine^38^, pyruvic acid^38^, l-lactic acid^38^, acetone^38^ and branched chain amino acids (valine, leucine, isoleucine)^28^ as cerebrospinal fluid biomarkers. Notably, in selected research projects, the adopted targeted metabolomic analysis proved to be helpful in differentiating cerebral infarction (CI) patients and those with intracerebral hemorrhage (ICH)^15,30^. Of course, the discriminatory power of the above-listed metabolites varied; however, statistical significance was maintained in each case. Similarly, favorable results have been obtained when using metabolomics in the analysis of states of traumatic brain injury (TBI), although far fewer research projects have been conducted here. The performed studies have shown that proline^33^, phosphoric acid^33^, β-hydroxybutyric acid^33^, galactose^33^, creatinine^33^, valine^33^, linoleic acid^33^, arachidonic acid^33^, medium-chain fatty acids (decanoic and octanoic acids)^34^ and sugar derivatives including 2,3-bisphosphoglyceric acid^34^ in the blood; as well as propylene glycol^35^, lactate^35,36^, glutamine^35^, creatine^35^ and glutamate^36^ in the cerebrospinal fluid should be considered potential markers of acute TBI and could even serve as death predictors^36^. As stated before, there are no reports in the literature on metabolomics in brain-dead patients; however, a few research projects using magnetic resonance spectroscopy and magnetic resonance imaging have been performed to study in vivo metabolic changes in brain tissue. The conclusions from these studies have been that high levels of lactate, choline and lipids and decreased levels of n-acetyl aspartate are prognostic factors of brain death^39–41^. It has also been demonstrated that ^31^P magnetic resonance spectra in these individuals are dominated by intense inorganic phosphate signals and are characterized by a complete absence of adenosine triphosphate (ATP) and phosphocreatine at the same time^42,43^.

Our results demonstrate that there are metabolites that can be considered potential biomarkers of brain death. The metabolomic serum analysis comparing brain-dead individuals to patients in coma revealed statistically significant increases in the concentrations of methanol, acetone, acetate and 3-methyl-2-oxovalerate and simultaneous statistically significant decreases in the concentrations of isoleucine, betaine, methylhistidine, glycine and valine. There were also significant changes in the concentrations of other metabolites that played significant roles in discrimination, although we were unable to identify them unambiguously (Table 3). All resonance signals underwent the primary identification procedure using statistical total correlation spectroscopy (STOCSY) and a two-dimensional NMR spectroscopic approach (analysis accuracy level 2 based on Sumer et al.^44^. The unidentified resonance signals were determined by their multiplicity patterns and chemical shifts. To precisely identify them, advanced methods of serum purification should be carried out, and then mass spectrometry (MS) analysis should be performed. We plan to accomplish these steps in the next stage of research.

It is not possible to compare the results of our research with the analyses of other authors, as no studies on metabolomics in brain death have been conducted so far. Moreover, we did not make comparisons with healthy people but with comatose patients, which prevents the results of our research from being compared to analyses known from the literature of other brain pathologies, where healthy individuals were always the reference points. As a result, a lower concentration of an individual metabolite in our comparisons does not exclude its being higher than in healthy persons and vice versa. In the case of brain death, it should also be noted that this condition affects not only brain tissue but also the functioning of the entire organism in a more extensive way than does any other brain pathology.

Medicine does not have any laboratory tests that are able to confirm brain death, so this condition is diagnosed only on the basis of clinical examination, optionally complemented by instrumental methods, which are not always easily accessible. From this point of view, the results of our study demonstrating the potential of ^1^H NMR-based metabolic serum fingerprinting with multivariate metabolomic data analysis are particularly valuable. Further studies in this field should not only be regarded as constituting a great scientific challenge but also as a necessity for modern medicine, especially intensive care and transplantation medicine.

## Supplementary Information

Supplementary Information.

## Data Availability

The datasets generated and/or analyzed during the current study are available from the corresponding author on reasonable request.

## Abbreviations

1D: One-dimensional
2D: Two-dimensional
ATP: Adenosine triphosphate
AUC: Area under the curve
BCAAs: Branched chain amino acids
BD: Brain death
CAC: Citric acid cycle
CI: Cerebral infarction
COSY: Correlation spectroscopy
COW: Correlation optimized warping algorithm
CP: Coma patients
CV: Coefficient of variation
CV-ANOVA: Analysis of variance of the corss-validated residuals
FDR: False discovery rate
GC: Gas chromatography
HSQC: ^1^H–^13^C heteronuclear single quantum correlation
ICH: Intracerebral hemorrhage
LC: Liquid chromatography
MS: Mass spectrometry
NMR: Nuclear magnetic resonance
NSE: Neuron-specific enolase
OPLS-DA: Orthogonal partial least squares discriminant analysis
Par: Pareto scaling
PCA: Principal component analysis
PLS-DA: Partial least squares discriminant analysis
PQN: Probabilistic quotient normalization
ROC: Receiver operating characteristic
STOSCY: Statistical total correlation spectroscopy
TOCSY: Total correlation spectroscopy
UV: Unit variance scaling
QCV: Coefficient of quartile variation
