# Supplementary material for: Brain-dead and coma patients exhibit different serum metabolic profiles: preliminary investigation of a novel diagnostic approach in neurocritical care
Source: Sci Rep. 2021 Jul 30;11:15519. doi: 10.1038/s41598-021-94625-3 (PMC8324823; doi:10.1038/s41598-021-94625-3)
Supplement: Supplementary file 1 — Supplementary Information. [file 41598_2021_94625_MOESM1_ESM.docx]

**Online supplementary material**

Table S1. List of assignments of ^1^H NMR resonance signals with chemical shift, KEGG and HMDBI ID. Presented in alphabetical order list of compounds spin systems that were used in multivariate and univariate analysis.

| **No.** | **Metabolite** | **Chemical shift** | **Multiplicity** | **HMDB ID** | **KEGG ID** |
| --- | --- | --- | --- | --- | --- |
| 1 | 3-Hydroxybutyrate | 1.185 | (d) | HMDB00357 | C01089 |
| 2 | 3-Methyl-2-oxovalerate | 1.107 | (d) | HMDB00491 | C03465 |
| 3 | Acetate | 1.904 | (s) | HMDB00042 | C00033 |
| 4 | Acetoacetate | 2.265 | (s) | HMDB00060 | C00164 |
| 5 | Acetone | 2.218 | (s) | HMDB01659 | C00207 |
| 6 | Alanine | 1.461 | (d) | HMDB00161 | C00041 |
| 7 | Betaine | 3.25 | (s) | HMDB00043 | C00719 |
| 8 | Chol/PC/GPC | 3.201 | (m) | HMDB00097; HMDB00284; HMDB00086 | C00114; C00588; C00670 |
| 9 | Citrate | 2.669 | (dd) | HMDB00094 | C00158 |
| 10 | Creatine | 3.029 | (s) | HMDB00064 | C00300 |
| 11 | Creatinine | 3.024 | (s) | HMDB00562 | C00791 |
| 12 | Formate | 8.441 | (s) | HMDB00142 | C00058 |
| 13 | Glucose | 3.232; 5.223 | (m); (d) | HMDB00122 | C00031 |
| 14 | Glutamate | 2.333 | (m) | HMDB00148 | C00025 |
| 15 | Glutamine | 2.427 | (m) | HMDB00641 | C00064 |
| 16 | Glycine | 3.54 | (s) | HMDB00123 | C00037 |
| 17 | π-M-hist/τ-M-hist | 7.024 | (s) | HMDB00479; HMDB00001 | C01152 |
| 18 | Methanol | 3.347 | (s) | - | - |
| 19 | π-M-hist/τ-M-hist | 7.726 | (s) | HMDB00479; HMDB00001 | C01152 |
| 20 | Isobutyrate | 1.055 | (d) | HMDB01873 | C02632 |
| 21 | Isoleucine | 0.991 | (d) | HMDB00172 | C00407 |
| 22 | L 1 | 0.856 | (m) | - | - |
| 23 | L 2 | 1.259 | (m) | - | - |
| 24 | L 3 | 1.555 | (m) | - | - |
| 25 | L 5 | 5.28 | (m) | - | - |
| 26 | L_4 + NAC | 2.027 | (m) | - | - |
| 27 | Lactate | 1.313; 4.101 | (d); (q) | HMDB00190 | C00186 |
| 28 | Leucine | 0.945 | (t) | HMDB00687 | C00123 |
| 29 | Mannitol | 3.67; 3.785 | (m); (s) | HMDB00765 | C00392 |
| 30 | Mannose | 5.171 | (d) | HMDB00169 | C00159 |
| 31 | Phenylalanine | 7.406 | (m) | HMDB00159 | C00079 |
| 32 | Pyruvate | 2.358 | (s) | HMDB00243 | C00022 |
| 33 | Tyrosine | 6.875 | (m) | HMDB00158 | C00082 |
| 34 | Unk 1 | 1.158 | (d) | - | - |
| 35 | Unk 2 | 1.169 | (t) | - | - |
| 36 | Unk 3 | 1.284 | (d) | - | - |
| 37 | Unk 4 | 1.913 | (m) | - | - |
| 38 | Unk 5 | 2.06 | (s) | - | - |
| 39 | Unk 6 | 2.148 | (s) | - | - |
| 40 | Unk 7 | 2.903 | (m) | - | - |
| 41 | Unk 8 | 3.164 | (s) | - | - |
| 42 | Unk 9 | 7.138 | (d) | - | - |
| 43 | Valine | 0.969 | (d) | - | - |

**
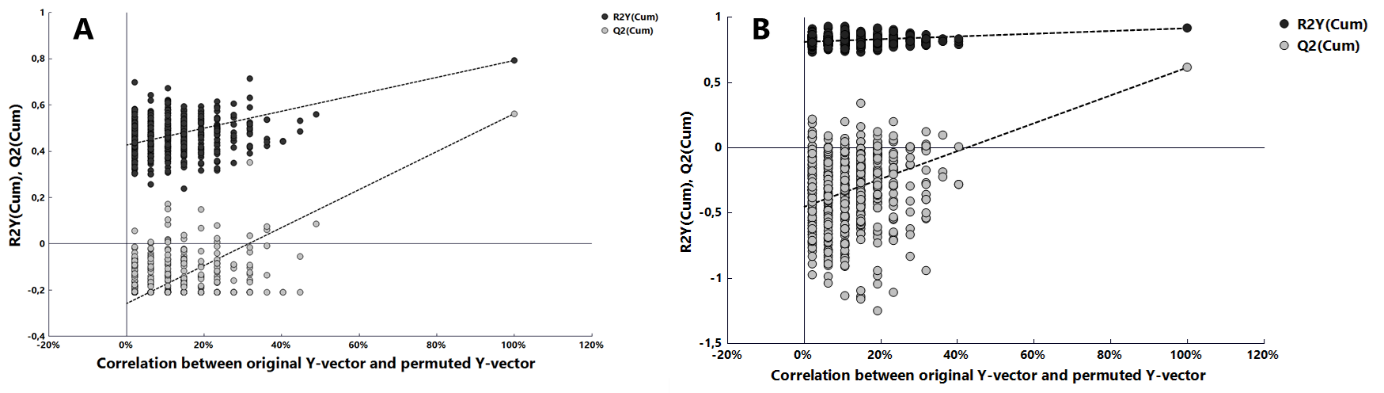
**

Figure S1. Response permutation test plots for (A) the PLS-DA model (Table 2, Figure 2) and (B) OPLS-DA (Table 2, Figure 3) calculated in study; 500 permutations were used. Drawing prepared in SIMCA 15.0.2.5959 (Sartorius Stedim Data Analytics AB, 2018).


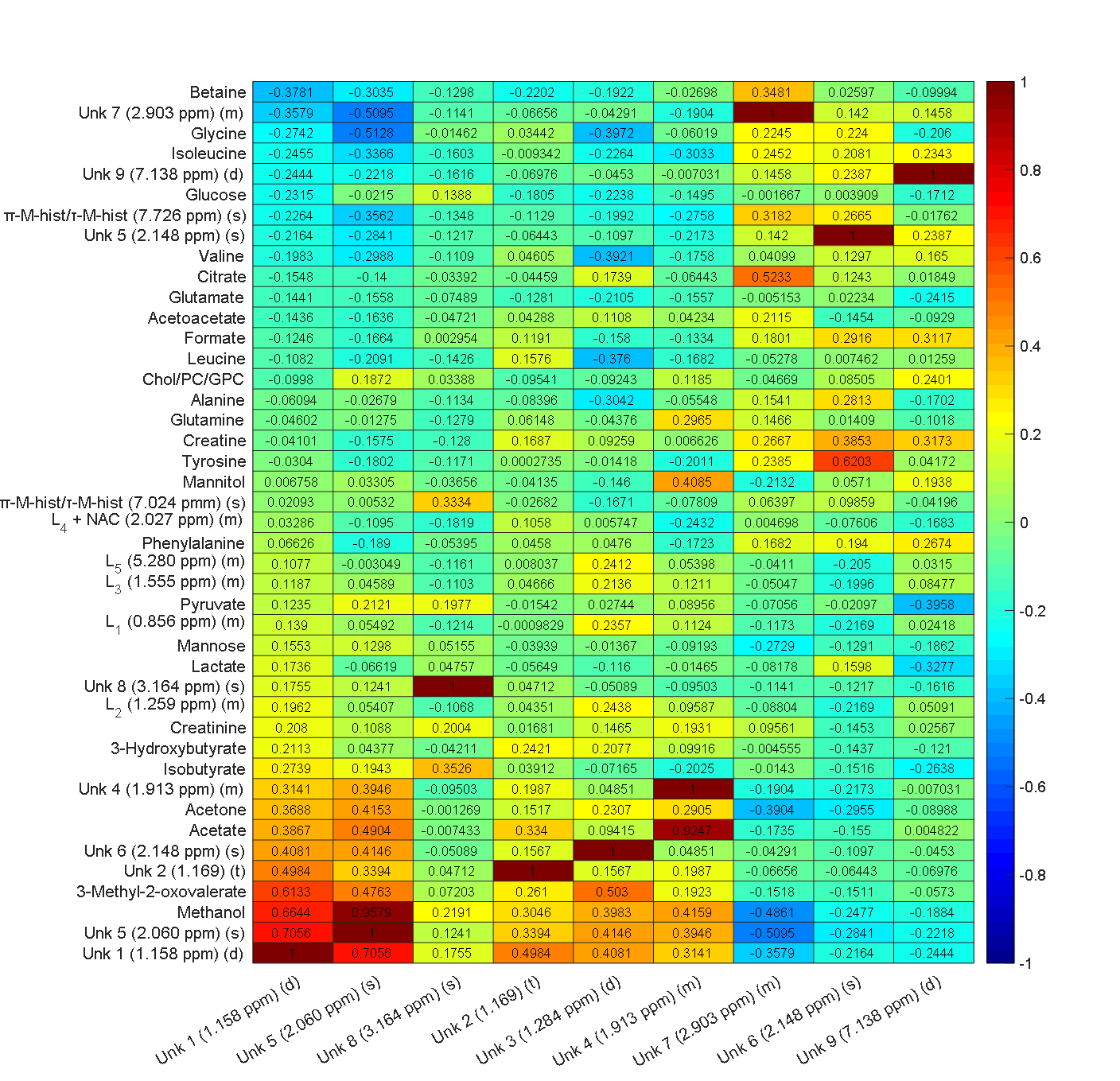


Figure S2. Pearson correlation heatmap for unassigned resonance signals for resonance signals identification. Gradient color between red and blue correspond to numerical values of Person rho (Dark red: 1.00, dark blue: -1.00). Drawing prepared in MATLAB (v R2019a, Mathworks Inc.).


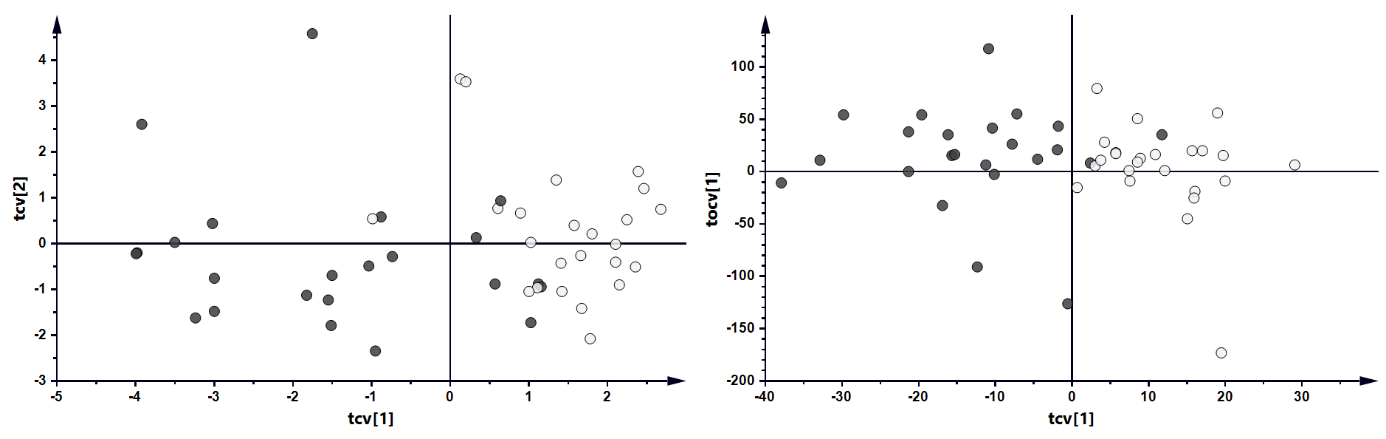


Figure S3. Cross-validated score plots for PLS-DA and OPLS-DA for verification of groups separation. Light gray – coma patients (CP); gray – brain-dead individuals (BD); Drawing prepared in SIMCA 15.0.2.5959 (Sartorius Stedim Data Analytics AB, 2018).
